# Cutaneous Chikungunya in a Nonfebrile Traveler to Madagascar

**DOI:** 10.4269/ajtmh.25-0543

**Published:** 2025-11-25

**Authors:** Naseem Alavian, Sofia Zavala, Robert Rolfe

**Affiliations:** ^1^ Division of Infectious Diseases, Department of Medicine, Duke University Medical Center, Durham, North Carolina;; ^2^ Duke Global Health Institute, Duke University, Durham, North Carolina

A 33-year-old female developed a diffuse pruritic rash 2 weeks into a research trip to Madagascar involving direct contact with lemurs. The rash began on her legs and spread to extremities, trunk, and face. She reported paresthesias that made ambulation uncomfortable but denied fever, arthralgia, abdominal pain, or diarrhea. Her only medication was atovaquone/proguanil for malaria prophylaxis.

Multiple coalescent pink macules, some with dusky center and targetoid appearance were seen on face, trunk, and extremities on day 7 of illness (Figure 1A and B). Confluent erythema involved bilateral soles with bullae on dorsal toes (Figure 1C). A single aphthae-like lesion was seen on the lingual frenulum.

**Figure 1.**
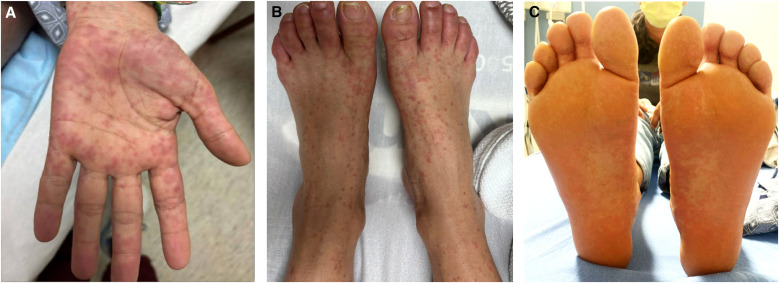
(**A** and **B**) Day 7 of illness maculopapular rash on hands and feet. (**C**) Confluent erythema on soles of feet with bullae on hallux.

Punch biopsy was performed prior to infectious disease specialist consultation. Histopathology revealed numerous dyskeratotic keratinocytes at all epidermal levels, concentrated in the superficial epidermis, coalescing into superficial necrosis (Figure 2).

**Figure 2.**
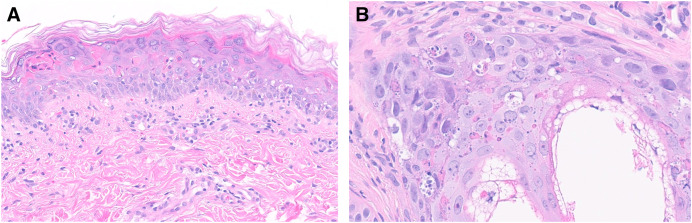
(**A**) Hematoxylin and eosin–stained slide showing numerous dyskeratotic keratinocytes present at all levels of the epidermis, accentuated at the epidermal surface and associated mild superficial lymphocytic infiltrate. (**B**) Dyskeratotic keratinocytes present in hair follicle epithelium with associated nuclear breakdown.

Chikungunya virus (CHIKV) IgM and IgG qualitative enzyme-linked immunosorbent assay testing were both positive on day 7 of illness. *Treponema pallidum* IgG, Dengue virus IgG/IgM and NS1 antigen, and Rocky Mountain spotted fever IgG were negative. The rash resolved over 4 weeks, with residual desquamation of the palms and soles at day 25 of illness (Figure 3).

**Figure 3.**
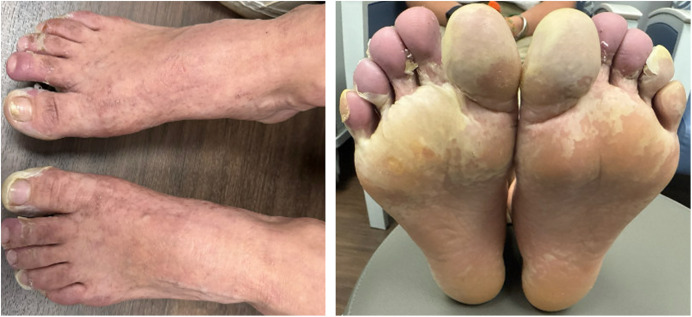
Day 25 of illness desquamation of bilateral feet.

Chikungunya is an arbovirus infection transmitted by *Aedes* mosquitoes, a vector that also transmits dengue, Zika, and yellow fever viruses.^1^ Fever and joint pains are generally predominant symptoms; however, a diverse range of cutaneous manifestations, including maculopapular rash, targetoid lesions, morbilliform rash, vesicles, bullae, and desquamation may occur.^2^ Recognition of these features, even in the absence of fever, is critical in returning travelers. In 2025, there have been outbreaks of chikungunya in countries in the Indian Ocean, including Madagascar.^3^ Madagascar is endemic for dengue as well but not known to be endemic for Zika.^3^ Climate change and increasing temperatures will likely contribute to expanded range of the vectors for CHIKV and other mosquito-borne arboviruses, with experts anticipating increase in autochthonous CHIKV transmission in North America.^4^^,^^5^

## References

[1] Rolfe RJ Zavala S Blackwood ER LaRocque RC Ryan ET, 2025. Mosquito-borne infections in international travelers. Wilderness Environ Med 36: 572–585. 40686432 10.1177/10806032251356485

[2] Oliveira JLD Nogueira IA Amaral JK Campos LR Mendonça MMM Ricarte MB Cavalcanti LPG Schoen RT, 2023. Extra-articular manifestations of Chikungunya. Rev Soc Bras Med Trop 56: 0341. 38088664 10.1590/0037-8682-0341-2023 PMC10706034

[3] US Centers for Disease Control and Prevention. 2025. *Travelers’ Health Madagascar*. Available at: https://wwwnc.cdc.gov/travel/destinations/traveler/none/madagascar. Accessed October 7, 2025.

[4] de Souza WM, , 2024. Chikungunya: A decade of burden in the Americas. Lancet Reg Health Am 30: 100673. 38283942 10.1016/j.lana.2023.100673 PMC10820659

[5] Delrieu M Martinet JP O’Connor O Viennet E Menkes C Burtet-Sarramegna V Frentiu FD Dupont-Rouzeyrol M, 2023. Temperature and transmission of chikungunya, dengue, and Zika viruses: A systematic review of experimental studies on *Aedes aegypti* and *Aedes albopictus*. Curr Res Parasitol Vector Borne Dis 4: 100139. 37719233 10.1016/j.crpvbd.2023.100139 PMC10500480

